# Differences in white matter detected by ex vivo 9.4 T MRI are associated with axonal changes in the R6/1 model of Huntington’s disease

**DOI:** 10.1016/j.nbd.2026.107318

**Published:** 2026-02-11

**Authors:** C. Casella, B. Kelly, A. Murillo, I. Mills-Smith, G.D. Parker, C. Von Ruhland, Y.A. Syed, V. Dion, C. Metzler-Baddeley, A.E. Rosser, D.K. Jones, M.J. Lelos

**Affiliations:** a Cardiff University, Brain Research Imaging Centre (CUBRIC), School of Psychology, Cardiff, UK; b School of Biosciences, Cardiff University, Cardiff CF10 3AX, UK; c UK Dementia Research Institute at Cardiff University, Hadyn Ellis Building, Cardiff CF24 4HQ, UK; d B.R.A.I.N Unit, Neurosciences and Mental Health Institute, School of Medicine, University Hospital of Wales, Heath Park, Cardiff CF24 4HQ, UK

**Keywords:** Diffusion MRI, Quantitative magnetization transfer, Huntingtons disease, White matter microstructure, R6/1 mice, Ultra-high field MRI

## Abstract

White matter volume loss has been reported as one of the first indicators in Huntington’s disease (HD) patients, but the cellular basis of this deficit remains to be elucidated. To address this, we assessed white matter micro-structure in the transgenic R6/1 mouse model of HD with ex vivo MRI. Specifically, a tractometry approach was employed to inspect region-specific differences across the corpus callosum (CC), while voxel-based morphometry (VBM) and tract-based spatial statistics (TBSS) were used to identify brain-wise white matter macro- and microstructure abnormalities. Alterations in the macromolecular proton fraction (MPF) from quantitative magnetization transfer (qMT) and in the intra-axonal signal fraction (FR) from the composite hindered and restricted model of diffusion (CHARMED) suggested regional decreases in myelin content and widespread increases in axonal density in this mouse model of HD. A cohort of sex/age-matched mice was assessed on cognitive and simple motor tasks to demonstrate that functional impairments were coincident with imaging deficits. Finally, the neurobiological basis of the MRI phenotype was assessed with histological and electron microscopy analyses in a cohort of sex/age-matched mice, revealing disruptions in axonal morphology (i.e. less complex, thinner axons) and organization (i.e. more densely packed axons) in this mouse model of HD. Furthermore, our results indicate that, at least early in disease progression, R6/1 mice present a reduction in the expression or content of myelin-associated proteins without significant alterations in the structure of myelin sheaths. Crucially, our findings highlight the potential of FR, an in vivo estimate of axon density, as a novel MRI biomarker of HD-associated changes in white matter microstructure.

## 1 Introduction

Huntington disease (HD) is an autosomal dominant neurodegenerative disorder characterised by progressive cognitive, psychiatric, and motor symptoms. Although symptomatic treatments are available, there is currently no cure and no approved disease-modifying therapy, making it a priority to clarify early disease mechanisms and develop biomarkers that can support therapeutic discovery and evaluation. Accumulating evidence indicates that alterations in brain white matter are a relevant feature of HD pathophysiology (Bardile et al., 2019; Bartzokis et al., 2007; Casella et al., 2020a; Casella et al., 2021; Casella et al., 2022; Casella et al., 2020b; Ciarmiello et al., 2006; Gregory et al., 2018; Paulsen et al., 2008; Reading et al., 2005; Rosas et al., 2018; Wang and Yang, 2019).

White matter atrophy has been demonstrated in both animal models and in human HD carriers by histopathological post-mortem studies (de la Monte et al., 1988; Gatto and Weissmann, 2019; Halliday et al., 1998; Huang et al., 2015; Jin et al., 2015) and MRI studies (Bartzokis et al., 2007; Casella et al., 2021; Casella et al., 2022; Rosas et al., 2018; Jin et al., 2015; Meng et al., 2017; Rosas et al., 2006; Tabrizi et al., 2009; Tabrizi et al., 2011). Structural neuroimaging studies of HD patients have reported widespread white matter loss across multiple tracts, including the corpus callosum, anterior commissure, internal and external capsules, and cingulum. Importantly, converging evidence indicates that white matter changes emerge early in the disease course (Ciarmiello et al., 2006; Tabrizi et al., 2009; Aylward et al., 2011; Di Paola et al., 2014; Ruocco et al., 2008; Humbert and Barnat, 2022). The severity of white matter atrophy correlates with predicted time to symptom onset in premanifest individuals (Ciarmiello et al., 2006; Paulsen et al., 2008; Stoffers et al., 2010), as well as with motor impairment (Rosas et al., 2010) and cognitive deficits (Rosas et al., 2006; Bohanna et al., 2008). Together, these findings motivate more mechanistic work to understand how white matter pathology develops and how it contributes to disease progression.

However, the biological basis of white matter degeneration in HD remains incompletely understood. Notably, white matter is composed of axons as well as myelin-producing oligodendrocytes, and it is unclear whether it is the loss of axons, myelin, or both, that drives the white matter volumetric loss (Gregory et al., 2018). A key limitation is that white matter volume derived from structural MRI is a relatively non-specific marker: apparent atrophy can arise from multiple processes, including Wallerian degeneration, reduced myelin content, changes in extracellular space, or mixed pathology. As a result, volumetric measures alone provide limited insight into the cellular substrates driving disease-related change.

Although diffusion tensor MRI (DT-MRI) (Basser et al., 1994) has allowed a deeper understanding of white matter organization at the microstructural level, DT-MRI metrics do not map specifically onto biological subcomponents of white matter microstructure (De Santis et al., 2014). It is therefore very hard to interpret changes in DT-MRI metrics in terms of specific microstructural properties. Very different configurations of, for example, axonal packing, axonal size, and myelination may generate very similar outcome measures (See Fig. 2 in (Harrison et al., 2020)). Accordingly, while some DT-MRI studies have suggested that white matter changes in HD are a consequence of axonal injury rather than demyelinating mechanisms (Weaver et al., 2009), several others have indicated a role for myelin disturbance in HD pathology (Bartzokis et al., 2007; Rosas et al., 2018; Di Paola et al., 2014; Bourbon-Teles et al., 2019; Di Paola et al., 2012; Mascalchi et al., 2004). This uncertainty highlights the need for microstructure-sensitive approaches with improved biological specificity.

In this work, we moved beyond volumetric and DT-MRI methods to better probe the biological substrates of white matter alterations reported in HD. We carried out a comprehensive microstructural assessment of the R6/1 mouse model of HD (Mangiarini et al., 1996; Naver et al., 2003) using 9.4 T MRI. We complemented gross measurements of white matter atrophy performed with voxel-based morphometry (VBM), and fractional anisotropy (FA), axial diffusivity (AD) and radial diffusivity (RD) from DT-MRI (Pierpaoli and Basser, 1996), with measures providing increased sensitivity to tissue microstructure and biochemical composition: the macromolecular proton fraction (MPF) from quantitative magnetization transfer (qMT) (Henkelman et al., 1993), and the restricted diffusion signal fraction (FR) from the composite hindered and restricted model of diffusion (CHARMED) (Assaf and Basser, 2005).

MPF quantifies the relative size of the semi-solid macromolecular pool exchanging magnetization with free water; in white matter, this pool is dominated by myelin, making MPF a widely used quantitative proxy for myelin content (Mancini et al., 2020). Consistent with this, EM estimates indicate that myelin comprises a substantial fraction of mouse corpus callosum tissue (~36–44% across subregions) (Sepehrband et al., 2015), and MPF has been validated against myelin histology in animal models, including cuprizone demyelination/remyelination where it tracks myelin loss and recovery, and correlates strongly with MBP-based measures (Khodanovich et al., 2019; Khodanovich et al., 2017). FR represents the fraction of the diffusion-weighted signal attributed to a restricted compartment and is commonly interpreted as a proxy for axon density (De Santis et al., 2014). Together, MPF and FR provide complementary sensitivity to myelin and axonal microstructure, allowing us to test whether R6/1 white-matter alterations are more consistent with demyelination, axonal loss/altered axonal organization, or a combination of both.

The R6/1 mouse model expresses exon 1 of the human HD gene with around 115 CAG repeats, and develops a progressive phenotype with early-onset neuropathology and reduced lifespan (Rattray et al., 2013). Behavioural impairments in motor and cognitive domains emerge from about 2 months of age (Assaf and Basser, 2005), alongside mHTT accumulation and brain atrophy (Rattray et al., 2013). Molecular and synaptic abnormalities also emerge early in this mouse model, including transcriptional dysregulation and synaptic pathway disruption that correlate with behavioural decline (Hodges et al., 2008; Gallardo-Orihuela et al., 2019; Smith et al., 2005; Torres-Peraza et al., 2008; Cummings et al., 2006; Mazarakis et al., 2005), and neurotrophic/neurogenic alterations such as reduced striatal BDNF and impaired hippocampal neurogenesis (Gharami et al., 2008; Spires et al., 2004; Grote et al., 2005; Rodriguez-Lebron et al., 2005). As a fragment-transgenic line, R6/1 primarily models toxic gain-of-function driven by aggregated exon-1 HTT fragments rather than the full pathogenic spectrum of HD (e.g., loss of wild-type huntingtin function). Nonetheless, R6/1 remains widely used in mechanistic and preclinical studies, making it important to characterise which cellular processes underlie its early white matter phenotype and how these relate to functional impairments.

In parallel, knock-in models (e.g., zQ175, Hdh) incorporate expanded CAG repeats into the endogenous murine Htt locus and provide a more genetically faithful representation of the disease. Longitudinal diffusion MRI in heterozygous zQ175DN mice demonstrates progressive white matter abnormalities (including reduced fibre density and cross-section) prior to overt neurodegeneration, with minimal evidence of myelin loss, implicating axonal or organisational disturbances rather than frank demyelination (Vidas-Guscic et al., 2024). Consistent with this, diffusion abnormalities in the anterior corpus callosum have been reported early in Ki140CAG mice, preceding changes in neuro-transmitter signalling or cortico-striatal atrophy (Pérot et al., 2022). Nevertheless, knock-in models generally exhibit slower, later-onset pathology than transgenic lines like R6/1, which can make early structural changes more difficult to detect without highly sensitive techniques.

Beyond the R6/1 and knock-in models discussed above, several other transgenic and knock-in rodent lines have been examined using diffusion MRI and related microstructural imaging methods, revealing alterations in axonal and myelin-related metrics at early disease stages. A recent review (Pérot et al., 2024), synthesises these findings and underscores the importance of microstructure-sensitive MRI for probing HD pathology in preclinical systems.

Despite growing evidence for grey matter pathology in R6/1 mice (Rattray et al., 2013; Gatto et al., 2021), white matter microstructure in the R6/1 model remains relatively under-characterised, particularly using MRI approaches that can help disentangle axonal versus myelin contributions. Early studies reported no detectable differences in corpus callosum volume at 9 or 17 weeks (Rattray et al., 2013), suggesting that gross atrophy may lag behind microstructural change. More recent ex vivo MRI by Gatto et al. identified early callosal microstructural alterations at 11 weeks despite preserved volume (Gatto et al., 2021), but the cellular basis of these white-matter changes was not directly assessed. Clarifying whether early white-matter abnormalities reflect axonal alterations, myelin-related changes, neuroinflammation, or a combination of these is important for interpreting both disease mechanisms and treatment effects in this widely used model.

In the present study, we assessed both macrostructural and microstructural white matter changes using ex vivo MRI of 4-month-old mouse brains. Post-mortem imaging enabled longer scan times and reduced motion-related confounds, supporting higher spatial resolution and improved signal- and contrast-to-noise ratios (Holmes et al., 2017).

To relate structural findings to function, we used a separate cohort to characterise disease-associated functional impairments in this model at 4 months of age. Specifically, we assessed attentional and visuospatial processing using the 5-choice serial reaction time task (5-CSRTT), as well as a range of simple motor tasks.

Finally, to investigate the neurobiological basis of the imaging results, we analysed separate age- and sex-matched cohorts of mice using light microscopy and transmission electron microscopy across corpus callosum segments, quantifying axonal and myelin features including axon size, axon density, and myelin thickness. By leveraging the increased biological specificity obtained with this multi-modal approach, this work aimed to refine mechanistic interpretation of white matter alterations in R6/1 mice and to inform understanding of early white matter involvement in HD.

## 2 Materials and methods

### 2.1 Mice

All experimental procedures (Table 1) followed protocols in accordance with the United Kingdom Animals (Scientific Procedures) Act of 1986. All experimental procedures performed on mice were approved by Cardiff University Ethical Review Process Committee and carried out under Home Office License P49E8C976.

We regularly undertook power calculations to ensure we would be sufficiently well-powered to detect differences across our assays. Effect sizes for performance on the behavioural task were based on previous in-house data collected in the lab (Reaction Time. Mean ± SD WT: 1.2 ± 0.7, R6/1: 2.5 ± 0.7), suggesting an effect size d of 1.857143, using G*Power. Based on a probability threshold of 0.05 and being powered to 0.80, a sample size calculator indicated a requirement for *n =* 5/group. For imaging and histology, we used previously published data assessing the integrity of the CC in R6/1 mice, where *n =* 4/group was found to be sensitive (Gatto et al., 2021).

Twelve (12-week-old) female hemizygous R6/1 mice and twelve wildtype littermates were purchased from Jackson Laboratories (Jax®, Bar Harbour, Maine, U.S.A.) and were scanned at 16 weeks of age. Only female mice were included to reduce potential confounds related to sex differences. For example, sex differences in weight, social behaviours and habituation have been reported in R6/1 mice (Harrison et al., 2013; Pietropaolo et al., 2011; Brooks et al., 2012). To ensure the accuracy and reliability of the analyses, all datasets underwent thorough quality control, and some were excluded due to artifacts or anomalies observed during imaging and preparation. In particular, certain specimens displayed air bubbles within the brain tissue, likely introduced during the perfusion fixation process. These bubbles caused localized disruptions in tissue integrity, appearing as signal voids or distortions in the imaging data (Supplementary Fig. 1). As a result, the final MRI dataset included 7 wildtype and 8 R6/1 mice, with only samples free from artifacts or degradation retained for further quantitative analysis (Cahill et al., 2012).

An age- and sex-matched cohort of R6/1 and WT littermate mice was used for electron microscopy (*N =* 3 WT and N = 3 R6/1). For light microscopy, an age- and sex-matched cohort that consisted of *N =* 9 WT and N = 9 R6/1 was used, but sections from a 1:12 series were only included for analysis if they fell within the boundaries determined for the genu, body and splenium (see Section 2.5).

For motor and cognitive testing, an age- and sex-matched cohort of mice consisting of WT (*N =* 6) and R6/1 (*N =* 5) was used. These mice were initially part of a separate study and injected with a control viral vector at P2, but subsequent to the behavioural testing at 4 months, it was found that expression of a dCas9 control vector expression was diminished after a few weeks; thus, behavioural data from these mice were used to assess cognitive and motor deficits in R6/1 mice at 4 months of age.

Prior to the experiment, all animals were housed in age- and sex-matched groups of between 1 and 5 mice, with mixed genotypes. Mice were subject to a 12-h light:12-h dark cycle with controlled room temperature (21 ± 3 °C) and relative humidity (60 ± 3%). Each cage contained modest environmental enrichment including play tunnels and nesting material. All animals were weighed on a weekly basis to monitor general health.

### 2.2 Assessment of visuospatial and attention function in the 5-CSRTT, and simple motor function

Mice were trained on the 5-choice serial reaction times task (5-CSRTT), starting at 1 month of age. They were food restricted 5 days before training commenced and the 5-CSRTT testing concluded when mice were ~ 3 months old. The 5-CSRTT task requires the mouse to attend to the array until a stimulus light is briefly presented. Successful nose poke in the operandum where the stimulus flash occurred results in milkshake reward. Failure to poke in the correct hole results in a brief timeout, during which time the house light is presented briefly, before another trial commences. This task measures visuospatial attention during the interval prior to onset of the stimulus, when the mouse is required to attend to the 5-hole array to detect the flash of the stimulus light. During this same period, impulsive behaviours can be detected if mice respond inappropriately, rather than waiting for the stimulus presentation. Accuracy refers to the number of correct responses made from the total number of trials that commenced. The reaction time is the duration of time between the stimulus onset and the mouse successfully responding in the nose poke operandum. Omissions refer to trials in which the stimulus light flashes, but no response is made during the 10 s limited hold period. Impulsivity refers to the number of nose pokes that occur during the interval prior to stimulus onset. Total trials refers to the total number of trials successfully completed during the test session.

Training was conducted as described in (Yhnell et al., 2016) (see Fig. 3A). In brief, mice were initially trained to retrieve strawberry milkshake reward from the magazine, followed by training to respond in the central nose poke of the array. During the initial training stages, the stimulus light was maintained for 10s to support learning, and this was gradually reduced to 2 s, 1 s, 0.5 s and finally 0.25 s stimulus duration.

From 3 to 4 months old, mice were maintained on an ad lib diet and tested on a range of simple motor tests, as described in (Brooks et al., 2012). Four trials of balance beam were conducted, and data were averaged across the trials. Two trials of fixed rotarod (12 rotations per minute) were conducted and the data were analysed across these two trials. Data consisted of the mean latency to fall and the number of falls in a 60s period. Two trials of the accelerated rotarod were run and data were averaged across these two trials. Two trials of vertical pole were conducted, and data were averaged across these two trials. Locomotor activity was also tracked in the open field using Ethovison over 5 min. Finally, grip strength was assessed using the wire hang test for a maximum of 60s.

#### 2.2.1 Statistical analysis

All behavioural data were analysed using GraphPad Prism 9. The accuracy data from 5-CSRTT data were analysed by ANOVA with stimulus duration (10s–0.25 s) as a within-subjects factor and genotype as a between-subjects factor. The remaining measures (reaction time, impulsivity, omissions, and total trials) and all simple motor tests were analysed by independent subjects *t*-test.

### 2.3 Perfusion

For imaging and light microscopy, mice were terminally anaesthetised via intraperitoneal injection of 0.3 ml Euthatal and then perfused through the left ventricle with approximately 60 ml of phosphate buffered saline (PBS). This was followed by infusion with about 150 ml of 4% formaldehyde in PBS (pH 7.3) at a flow rate of 30 ml/min. The temperature of the perfusates was maintained at room temperature and the pH of the formaldehyde was 7.2–7.4. After decapitation, the skulls were de-fleshed and post-fixed in 4% formaldehyde in PBS over-night. They were then transferred to a 25% sucrose solution and stored at 4°. For electron microscopy, a similar procedure was followed, except that the perfusate contained 2.5% Glutaraldehyde +2.5% para-formaldehyde in PBS.

### 2.4 Ex vivo MRI assessment of R6/1 brains

#### 2.4.1 Tissue preparation

Brains were scanned in-skull, to preserve neural structures. The skulls were soaked in PBS and washed daily for three days, to regain some signal due to tissue rehydration (Petiet et al., 2011). The skulls were then carefully wiped with tissue paper, and immersed in Galden, a proton-free susceptibility-matching fluid in a 15 ml syringe. The use of a syringe allowed any residual air bubbles to be pushed out, which might otherwise have affected MRI measurements. Immediately after scanning, skulls were returned to PBS and washed for three days, before being stored in a 25% sucrose - 0.1% sodium azide solution at 4°, to ensure tissue preservation.

#### 2.4.2 Data acquisition

MRI acquisition was conducted ex vivo on a 9.4 Tesla (20 cm) horizontal-bore animal system (Bruker Biospin, Germany). This was equipped with BGA12-S (12 cm inner bore size, integrated shims) gradients. A transmit 1H 500 W echo-planar imaging (EPI) volume coil was used with a phased array 4-channel surface coil and Paravision software (version 6.1, Bruker Biospin) were used for data acquisition.

The magnetic field homogeneity was optimized with a localized shimming procedure (Fastmap, Bruker Biospin) on a volume of interest placed in the centre of the brain. A PRESS-waterline sequence (Bruker BioSpin) was used with outer volume suppression without water suppression to evaluate water line width (TR/TE = 2500/20 ms respectively) to assess the shim performance and the peak line width of the water signal. Iterations were repeated until all line widths <40 Hz.

The acquisition protocol consisted of a T_1_-weighted FLASH sequence, a multi-shell dMRI acquisition for DTI and CHARMED (Basser et al., 1994; Assaf and Basser, 2005), and an MT-weighted (MT-w) T_1_ FLASH sequence. Additionally, the longitudinal relaxation rate of the system was estimated by acquiring T_1_-maps using T_1_-weighted FLASH images. To maintain signal stability and minimize RF-induced heating, the RF pulse magnitudes were alternated to limit temperature-related fluctuations. A detailed description of the acquisition parameters for each sequence is provided in Table 2.

#### 2.4.3 Image processing

Skull-stripping was performed using the Rodent Bet Brain Extraction Tool, a modified version of the Brain Extraction Tool (BET; FSL v5.0) that can process rodent brains (Wood et al., 2013).

##### 2.4.3.1 Anatomical data

Processing of T_1_ anatomical images was performed using SPM8 (Wellcome Trust Institute of Neurology, University College London, UK, www.fil.ion.ucl.ac.uk/spm) with the SPMMouse toolbox (http://spmmouse.org) for animal brain morphometry (Sawiak et al., 2013). This toolbox extends SPM’s functionality with affine registration priors for mouse brains.

Specifically, a previously described mouse brain atlas (Sawiak et al., 2009) was used to register images from the brains of R6/1 mice with those of WT littermate controls. Following approximate manual registration using the SPM interface, images were bias-corrected, and the affine priors supplied were used to register the images to the tissue probability maps. The registered images were then used to obtain grey matter, white matter and CSF segmentations. The resulting white matter segmentations were output in rigid template space and DARTEL (Ashburner, 2007) was used to create both non-linearly registered maps for each subject, and common templates for the cohort of animals. The warped white matter portions for each mouse brain were modulated with the Jacobian determinant from the DARTEL registration fields to preserve tissue amounts, and smoothed with a Gaussian kernel of 400 μm to produce maps for analysis (Gary et al., 2019).

##### 2.4.3.2 Diffusion data

**Fig. 1:**
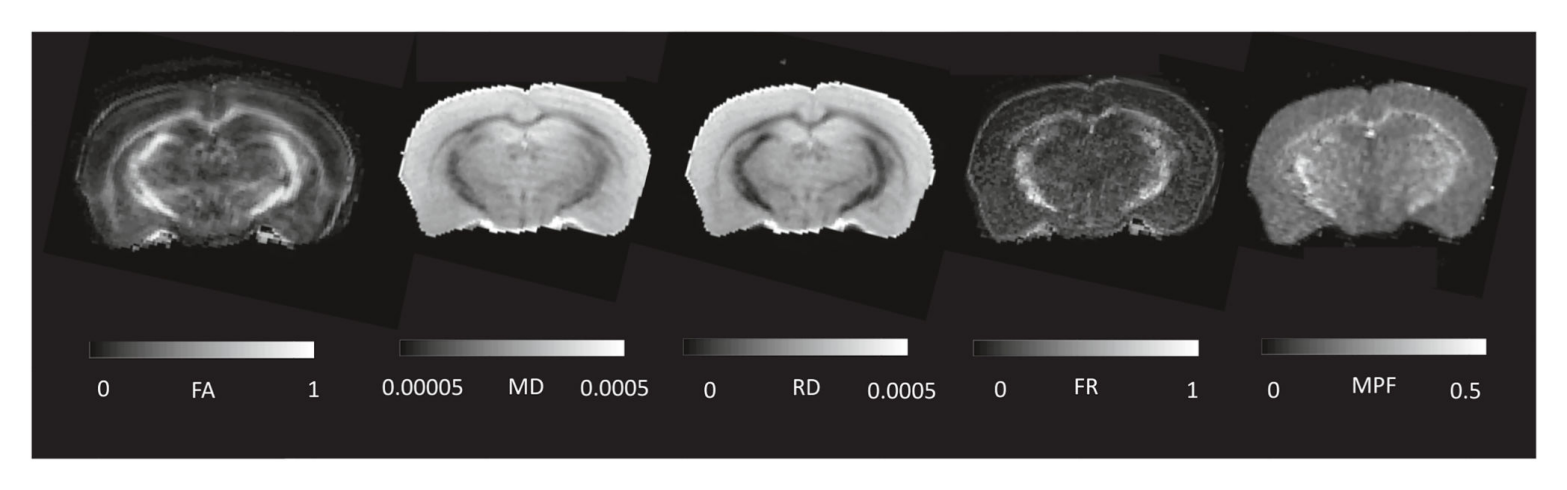
Parameter maps for a representative mouse in a coronal slice. The contrast observed across different maps provides complementary information on tissue composition and aligns with known brain microstructure.

Each DWI dataset was pre-processed as follows: denoising was performed using the method described in (Veraart et al., 2016) and implemented in the MRtrix3 (Tournier et al., 2019) software package; field distortions (Andersson et al., 2003) were corrected using the topup and eddy tools in FSL (Smith et al., 2004); and Gibbs ringing artifacts were mitigated using the mrdegibbs tool in MRtrix3 (Tournier et al., 2019), which applies a sub-voxel shift algorithm to enhance data quality while preserving spatial resolution (Kellner et al., 2016). Diffusion tensors were estimated using non-linearly weighted least squares (for b < 1500 s/mm^2^ data) providing the following quantitative scalar measures: FA, AD and RD. Motion and distortion artifacts in the CHARMED data were corrected according to the extrapolation method described in (Ben-Amitay et al., 2012). FR maps were computed by fitting the CHARMED model to the DWI data, with an in-house software coded in MATLAB (The MathWorks, Natick, MA). Representative parameter maps are shown in Fig. 1.

##### 2.4.3.3 qMT data

MT-w images were corrected for Gibbs ringing artifacts (Kellner et al., 2016) and co-registered to the MT-w volume with the most contrast using an affine registration (FLIRT, 12 degrees of freedom, mutual information). Subsequently, the MT-w images and T_1_-maps were modelled by the two-pool Ramani’s pulsed MT approximation (Ramani et al., 2002). This provided MPF maps (Fig. 1), which were nonlinearly registered to the b = 0 s/mm^2^ image using the MT-w volume with the most contrast as a reference, using FNIRT (Andersson et al., 2007). Accuracy of all registrations was confirmed by visual inspection.

#### 2.4.4 Tractography of the CC

Multi-shell, multi-tissue, constrained spherical deconvolution (MSMT-CSD) (Jeurissen et al., 2014) was applied to the pre-processed images to obtain voxel-wise estimates of the fibre orientation density functions (fODFs) (Descoteaux et al., 2008; Tournier et al., 2004; Tournier et al., 2007) with maximal spherical harmonics order lmax = 8. The fODFs were generated using a set of 3-tissue group-averaged response functions (Dhollander et al., 2016). Seed points were positioned at the vertices of a 0.2 × 0.2 × 0.2 mm grid superimposed over the image. The tracking algorithm interpolated local fODF estimates at each seed point and then propagated 0.05 mm along orientations of each fODF lobe. This process was repeated until the minimally subtending peak magnitude fell below 0.05 or the change of direction between successive 0.05 mm steps exceeded an angle of 40°. Tracking was then repeated in the opposite direction from the initial seed point. Stream-lines whose lengths were outside a range of 2 mm to 30 mm were discarded.

**Fig. 2:**
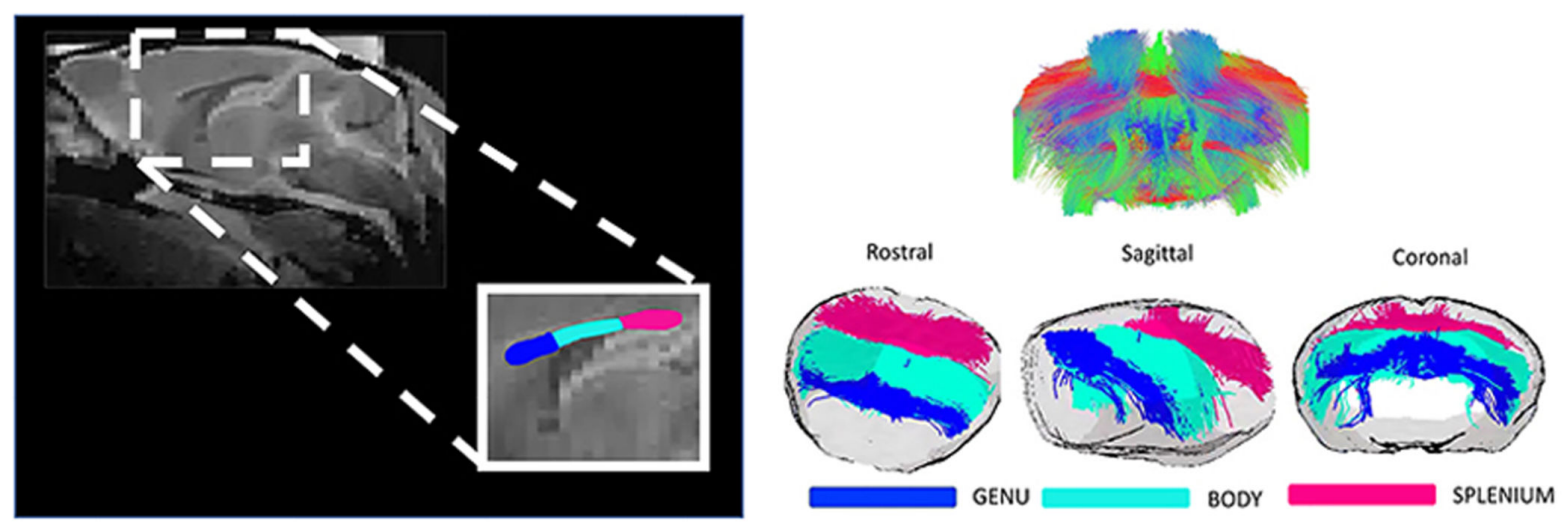
Tractography of the corpus callosum (CC). Left: Representative figure of manually delineated segments of the corpus callosum (genu, body, and splenium). Right: Coronal view of whole brain tractography (top) and fibres travelling through the manually delineated region of interests (ROIs), in rostral, sagittal and coronal views (bottom).

To assess regionally specific effect of HD on the corpus callosum, tractography was performed in three different callosal segments (genu, body and splenium). 3D fibre reconstructions were performed interactively in the native space of each mouse in FiberNavigator (Chamberland et al., 2014) (Fig. 2), using a combination of include and exclude regions of interest (ROIs), according to the following protocols:

##### 2.4.4.1 Genu

Two ROIs were placed anterolateral to the most rostral portion of the corpus callosum in each hemisphere. This approach was used to capture the anteriorly arching fibres of the genu (Catani and Thiebaut de Schotten, 2008). An exclusion ROI was used to exclude streamlines extending posteriorly to the genu on the sagittal plane, which make up the body of the corpus collosum.

##### 2.4.4.2 Body

Two ROIs were placed ventral to the location of the cingulum and medial to the lateral ventricles (one in each hemisphere) (Catani and Thiebaut de Schotten, 2008).Exclusion ROIs were used to exclude the genu and splenium (i.e., the anterior and posterior sections of the CC, respectively).

##### 2.4.4.3 Splenium

Two ROIs were placed posterolateral to the most caudal section on the corpus collosum in the left and right hemisphere (Catani and Thiebaut de Schotten, 2008). An exclusion ROI was used to remove streamlines extending anteriorly to the splenium.

#### 2.4.5 Statistical analysis

Statistical analyses were carried out using RStudio v1.1.456 (Team Rs, 2015), MATLAB (The MathWorks, Natick, MA), SPSS version 20,119 (Armonk and IBM Corp, 2011), FSL (Smith et al., 2004) and the SPMMouse toolbox (http://spmmouse.org) for animal brain morphometry (Sawiak et al., 2013).

##### 2.4.5.1

*Tractometry of the corpus callosum*. Microstructure differences were assessed in each of the three callosal segments. First, by taking each quantitative metric map (each registered to the b = 0 s/mm^2^ image during pre-processing), samples of each metric were obtained at each vertex of the reconstructed segments, and segment-specific medians were derived for FA, AD, RD, FR and MPF in MRtrix3 (Tournier et al., 2019). Next, the overall mean was calculated, so that each dataset comprised 5 MRI-derived measures, mapped along 3 callosal segments.

##### 2.4.5.2 Investigation of group differences in callosal microstructure

Two-way robust mixed ANOVAs were run for each metric (i.e., FA, RD, AD, FR, MPF), using the “bwtrim” R function from the WRS2 package (Mair and Wilcox, 2020). This implements robust methods for statistical estimation and therefore provides a good option to deal with data presenting small sample sizes, skewed distributions and outliers (Wilcox, 2011). Group was the between-subject factor and segment was the within-subject variable. Given that this robust method does not readily allow for post-hoc tests, we adopted a manual approach to further investigate significant effects. For each callosal segment, trimmed means were computed by removing the most extreme 20% of values (10% from each tail of the distribution) before calculating the mean. This approach reduces the influence of outliers and provides a more robust estimate of central tendency. After obtaining the trimmed means, pairwise comparisons were conducted with paired-samples *t*-tests, and *p*-values corrected for multiple comparisons using the false discovery rate (FDR). Across all analyses, outliers that were ±3 standard deviations from the mean were removed.

##### 2.4.5.3 Automatic evaluation of white matter atrophy using VBM

A general linear model to assess group differences in white matter volume was evaluated using SPMMouse (Sawiak et al., 2013). ICV was included as a covariate of no interest as this was shown to improve estimation of volume differences in previous literature (Sawiak et al., 2013). ICV was calculated as the sum of voxels identified as grey matter, white matter and CSF in native space for each animal, to model out the effect of different brain sizes. The sum of the white matter tissue probability maps was used as explicit mask in the analysis. An adjusted p-value was calculated to control the voxel-wise FDR (Benjamini and Hochberg, 1995).

##### 2.4.5.4 Assessment of brain-wise group differences in white matter microstructure using TBSS

To perform a whole-brain analysis of white matter microstructure changes associated with HD, the TBSS protocol (Smith et al., 2006), modified for use in rodents (Sierra et al., 2011) was used. All FA maps were submitted to a free search for a best registration target in order to minimize the image warping required for each brain volume. Specifically, each volume was first registered to every other volume, and the one requiring minimum transformation to be registered to other volumes was selected as the best registration target. This target was used as a template into which the registration was performed. Following registration, a mean FA map was calculated, thinned to represent a mean FA skeleton, and an optimal threshold of 0.2 was applied to the mean FA skeleton to create a binary white matter skeleton mask. The application of such threshold allowed to exclude from further analysis areas of the brain of low FA, including peripheral small tracts, where there may be high between-subject variability and grey matter, and it is therefore unsafe to assume good tract correspondence across subjects (Smith et al., 2006).

The local FA-maximum was projected onto this white matter skeleton. Subsequently, the voxel location of the local FA maximum was employed to project the respective AD, RD, FR and MPF values from that voxel onto the skeleton. Differences in microstructure measures between the two groups were assessed using voxel-wise independent *t*-tests (assessing areas where WT > R6/1 and R6/1 > WT). The randomize function (part of FSL) was used, together with the TFCE algorithm (Smith and Nichols, 2009), generating cluster-size statistics based on 1000 random permutations. For multiple comparison correction, FDR correction was used with a threshold of *p* < 0.05.

### 2.5 Cytoarchitectural assessment of the corpus callosum using immunohistochemistry

#### 2.5.1 Tissue processing

WT and R6/1 brains were frozen on a sledge-microtome (Leitz, Wetzlar), cut into 40 μm coronal sections, and collected in 12 parallel series. Sections were stored in ethylene glycol-based cryoprotectant at −20C until processing. For immunostaining, 1:12 series of sections were quenched for 5 min using 10% H_2_O_2_ (VWR, West Sussex, UK) and methanol (Sigma-Aldrich, Dorset, UK). Sections were blocked in 3% serum in Triton-X and Tris-buffered saline (TxTBS) for 1 h and incubated overnight, at room temperature in a solution of TxTBS, 1% serum and primary antibodies raised against either myelin basic protein (MBP; 1:1000, Santa Cruz, cat. # SC1394R), 68 kDa Neurofilament (NFL; 1:500, Abcam, cat. # ab72997), microglia (Iba1; 1:500; Wako, cat # 019–19,741) or astrocytes (GFAP; 1:1000, Dako, cat. # Z0334). Incubation in biotinylated-secondary antibody (1:200) was conducted for 2 h, then sections were incubated using an ABC kit (Vector Laboratories Ltd., Peterborough, Cambridgeshire) for 2 h. Proteins were visualised using 3–3′-diaminobenzadine (DAB), before mounting on to double-subbed 1% gelatinised slides (Thermo Scientific, Menzel Gläser). After dehydration and delipidisation in 100% xylene, slides were coverslipped using DPX mountant (Thermo Scientific, Raymond Lamb, Leicestershire, UK).

#### 2.5.2 Determination of corpus callosum regions of interest (ROIs)

To remain consistent with the DTI data, the corpus callosum was divided into three equal sections along its rostral-caudal axis to represent the genu, the body, and the splenium, mentioned rostral-caudal respectively. Bregma co-ordinates according to the Paxinos and Franklin Mouse Brain Atlas (Franklin and Paxinos, 2019) were used: genu 1.18 mm to 0.02 mm, body −0.1 mm to −1.22 mm and the splenium −1.3 mm to −2.54 mm. Three ROIs were taken from each brain hemisphere as well as 1 Medial ROI (Fig. 7A). For full brain slices, ROIs were placed in identical locations on each hemisphere where their quantifications were then averaged.

#### 2.5.3 Data collection

Bright field LM was used under identical conditions to visualise NFL and MBP immunoreactivity using the Olympus BX50 light microscope. All images of the genu, body and splenium were captured at 100× magnification with MicrosoftVIS software. The viewing field, intensity and aperture measurements remained consistent throughout imaging to ensure the data processing was indistinguishable. For image quantification, using ImageJ software (NIH, v 1.53r), all images were quantified according to two different measurements, corpus callosum thickness (μm) and area fraction (AF; i.e. the percentage of area with positive immunostaining). Images were converted to 8-bit greyscale prior to measurements being taken. For the analysis of the genu, body and splenium, all ROIs for each medial-lateral position were averaged together to determine a representative measurement (Fig. 7A).

In order to validate that our R6/1 mouse line is representative of this model within other laboratories, we used the NFL immunostained sections to estimate the size of the striatum in WT and R6/1 mice. We observed a mean volume and SEM of 22.99 ± 0.59 mm^3^ for WT striata and 18.63 ± 1.29 mm^3^ for R6/1 striata. This is approximately a 19% reduction in the mutant striata, which is consistent with the literature at this age (Rattray et al., 2013).

#### 2.5.4 Statistical analysis

Statistical analysis was conducted using IBM SPSS Statistics 27. ANOVAs were conducted on data collected along the mediolateral axis, with the between-subjects factor of Genotype (WT vs R6/1) and the within-subjects factor of Region (Medial, Central-Medial, Central-Lateral and Lateral).

### 2.6 Ultra-structural analysis of axons in the corpus callosum using transmission electron microscopy

#### 2.6.1 Tissue processing

1 mm thick slices in the central-medial region of the corpus callosum body segment were post-fixed for 2 h in 2% (*w*/*v*) aqueous osmium tetroxide, block stained for 2 h in 2% aqueous uranium acetate, dehydrated through graded isopropanol (50%, 70%, 90% and 2 × 100%) and 3 x propylene oxide for 15 min each and infiltrated with TAAB embedding resin (50% in propylene oxide, 4 x neat resin for 1 h each). Samples were placed into embedding moulds containing fresh resin and cured for 24 h at 60C.

For LM, semithin (0.5 μm) sections were collected onto droplet of distilled water on glass slides, dried on a hot plate, stained with 0.5% aqueous toluidine blue, and mounted in Gurr’s neutral mountant. Sections were examined with an Olympus BX51 research light microscope (Olympus Optical Co. (U.K.) Ltd., London, U⋅K) and images captured with a Zeiss Axiocam and Axiovision software (Carl Zeiss Vision GmbH, Hallbergmoos, Germany).

For electron microscopy, ultrathin (80-100 nm) sections were collected onto 300 mesh copper grids, stained with Reynolds lead citrate (Reynolds, E. S. (1963)) and examined in a Hitachi HT7800 TEM (Hitachi High Tech Ltd., UK) at 100 kV. Images captured with Radius software (EMSIS GmbH, Germany).

#### 2.6.2 Image quantification

Five regions within the body of the corpus callosum were taken for quantification from randomly sampled 16.193μm^2^ electron micro-graphs. The axon diameter of myelinated fibres and g-ratio of myelinated axons (calculated as ‘inner diameter’/’outer diameter’, where the inner diameter was that of the axon and the outer diameter include the myelin sheath) were manually quantified using ImageJ (https://imagej.net). Specifically, the inner and outer diameters were measured at the narrowest point of each axon, assuming them to be cylindrical.

#### 2.6.3 Statistical analysis

Statistical analysis was conducted using IBM SPSS Statistics 27. For the axonal data, an ANOVA was conducted with Genotype and Axon (myelinated, non-myelinated) as factors. For the remaining data, independent-samples *t*-tests were conducted with WT and R6/1 data.

## 3 Results

### 3.1 R6/1 mice present with early cognitive and motor impairments

**Fig. 3:**
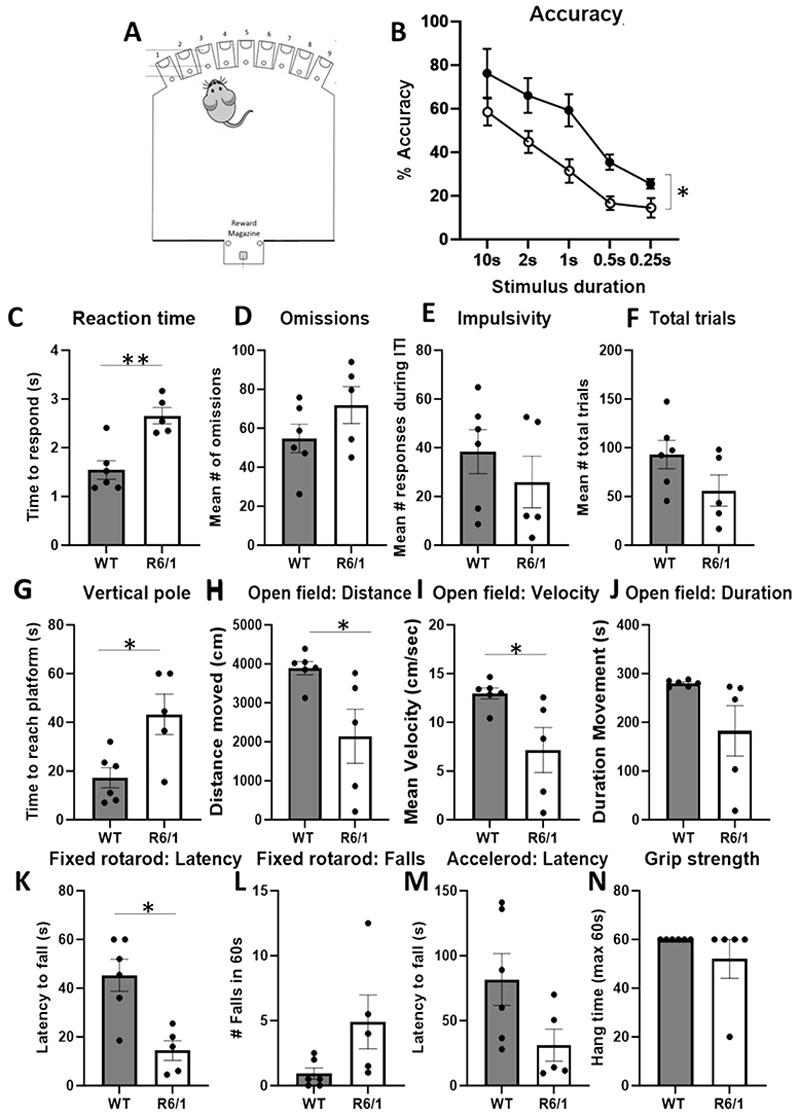
Analysis of cognitive and motor function in WT (*n* = 6) and R6/1 mice (*n* = 5). (A) Schematic showing the 9-hole operant box apparatus with every other nose poke hole available, a food reward magazine at the back of the box and a representative mouse. (B) Impaired response accuracy in R6/1 mice is evident on the 5-CSRTT, even when the response stimulus duration was long during training (e.g., 10s). On the 5-CSRTT, R6/1 mice also reacted to the stimulus more slowly (C), but no differences in response omissions (D), impulsivity, as assessed by the number of hole pokes during the inter-trial interval (E), or trials completed (F) was observed. R6/1 mice were slower on the vertical pole to reach the platform (G). In the open field, R6/1 mice travelled less far (H) and were slower (I), but spent a similar amount of time moving (J). On the fixed rotarod, R6/1 mice fell off more quickly (K) but did not fall more times across the 60s session (L). No differences in accelerod (M), nor grip strength on the hang wire test (N), were evident. Error bars = ±standard error of the mean (S.E.M.). *p ≤ 0.05, **p ≤ 0.01, ***p ≤ 0.001.

Functional assays were run to determine if 3–4-month-old R6/1 mice presented with cognitive or motor deficits at the age they underwent imaging and histological analyses. On the 5-CSRTT, R6/1 mice showed impaired accuracy to respond in the correct nose poke operandum across the training (e.g. 10s) and test sessions [Fig. 3B; F(1,9) = 7.228, *p* < 0.05]. R6/1 mice were slower than WT mice [Fig. 3C; t(9) = 4.288, *p* < 0.01], but no differences in impulsivity, response omission or trials completed were evident [Fig. 3D-F; max. t(9) = −1.707, p = n.s.]. R6/1 mice showed less dexterity than WT mice on the vertical pole test [Fig. 3G; t(9) = 2.971, p < 0.05]. On the open field test, R6/1 mice travelled less distance and were slower [Fig. 3H-I, min t(9) = −2.664, p < 0.05], and spent a similar amount of time moving compared to WT mice [Fig. 3J, min t(9) = −2.091, p = n.s.]. On the fixed rotarod, R6/1 mice fell more quickly [Fig. 3K; t(9) = −3.882, *p =* 0.01], but fell a similar number of times in the 60s session [Fig. 3L; t(9) = 2.064, p = n. s.]. No differences in grip strength were detected using the wire hang test nor in accelerod performance [Fig. 3M-N, max t(9) = −2.052, p = n. s.].

### 3.2 Tractometry analysis suggests that R6/1 mice present with alterations in apparent myelin, based on MPF, and axon density, based on FR, across the CC

**Fig. 4:**
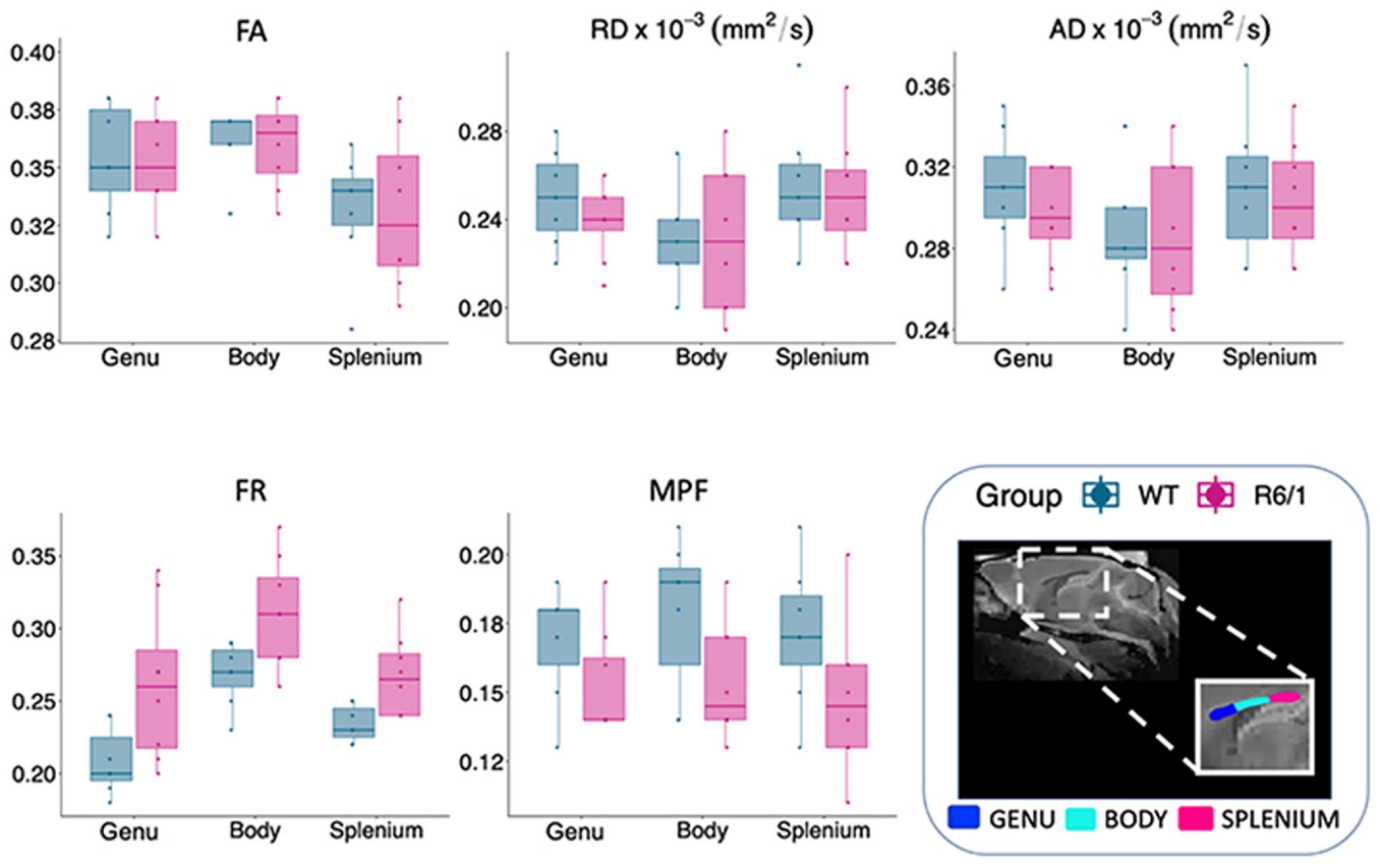
Tractometry analysis of the corpus callosum (CC). Quantification of FA, RD, AD, FR, and MPF in the genu, body and splenium of the CC. FR values were significantly higher and MPF values significantly lower in the brain of R6/1 mice (*n* = 8) across the whole CC, comapred to WT (*n* = 7). No significant group effects were detected for the other measures. Abbreviations: FA: fractional anisotropy; RD: radial diffusivity; AD: axial diffusivity; FR: restricted volume fraction; MPF: macromolecular proton fraction. The box plot displays the median, lower/upper quartile and minimum/maximum values.

Results for this analysis are plotted in Fig. 4. The mixed ANOVA for FA showed no significant effect of group [F(1, 7.4) = 0.31, *p* = 0.592]. On the other hand, a significant effect of segment [F(2, 6.05) = 7.51, *p =* 0.02] was detected, with the splenium showing significantly lower FA values compared to the body (*p =* 0.001). No interaction effect between group and segment was present [F(2, 6.05) = 0.009, *p =* 0.99].

The mixed ANOVA for RD showed no significant effect of group [Fig. 4; F(1, 7.2) = 0.16, *p =* 0.69], but a significant effect of segment [F (2, 6.4) = 15.15, *p =* 0.003], with the body showing significantly lower RD values compared to the splenium (*p* < 0.001) and the genu (*p=* 0.04). No interaction effect between group and segment was detected [F (2, 6.4) = 0.29, *p =* 0.75].

The assessment of AD values showed no significant effect of group [Fig. 4; F(1, 7.79) = 0.12, *p =* 0.73] but a significant effect of segment [F (2, 6.2) = 10.35, *p =* 0.01], with the body showing significantly lower AD compared to the genu (*p =* 0.009) and the splenium (p < 0.001).

The group-by-segment interaction was not significant [F(2, 6.2) = 0.44, *p =* 0.66].

Significant main effects of group [F(1, 5.47) = 7.21, *p =* 0.03] and segment [F(2, 6.36) = 43.57, p < 0.001] on FR values were detected with the mixed ANOVA. Specifically, R6/1 mice presented overall higher FR values compared to WTs across all corpus callosum regions. The corpus callosum body presented higher FR values compared to the genu (p < 0.001), the splenium presented lower FR values compared to body (p < 0.001). No significant group-by-segment interaction was detected [F(2, 6.36) = 0.24, *p =* 0.78].

Finally, a significant effect of group [F(1, 6.59) = 4.77, *p =* 0.05] was detected on MPF, with R6/1 mice showing significantly lower values. No significant effect of segment [F(2, 6.15) = 2.34, *p* = 0.17], nor a significant group-by-segment interaction effect [F(2, 6.15) = 0.12, *p =*0.88] were detected.

### 3.3 R6/1 mice present areas of increased white matter volume

**Fig. 5:**
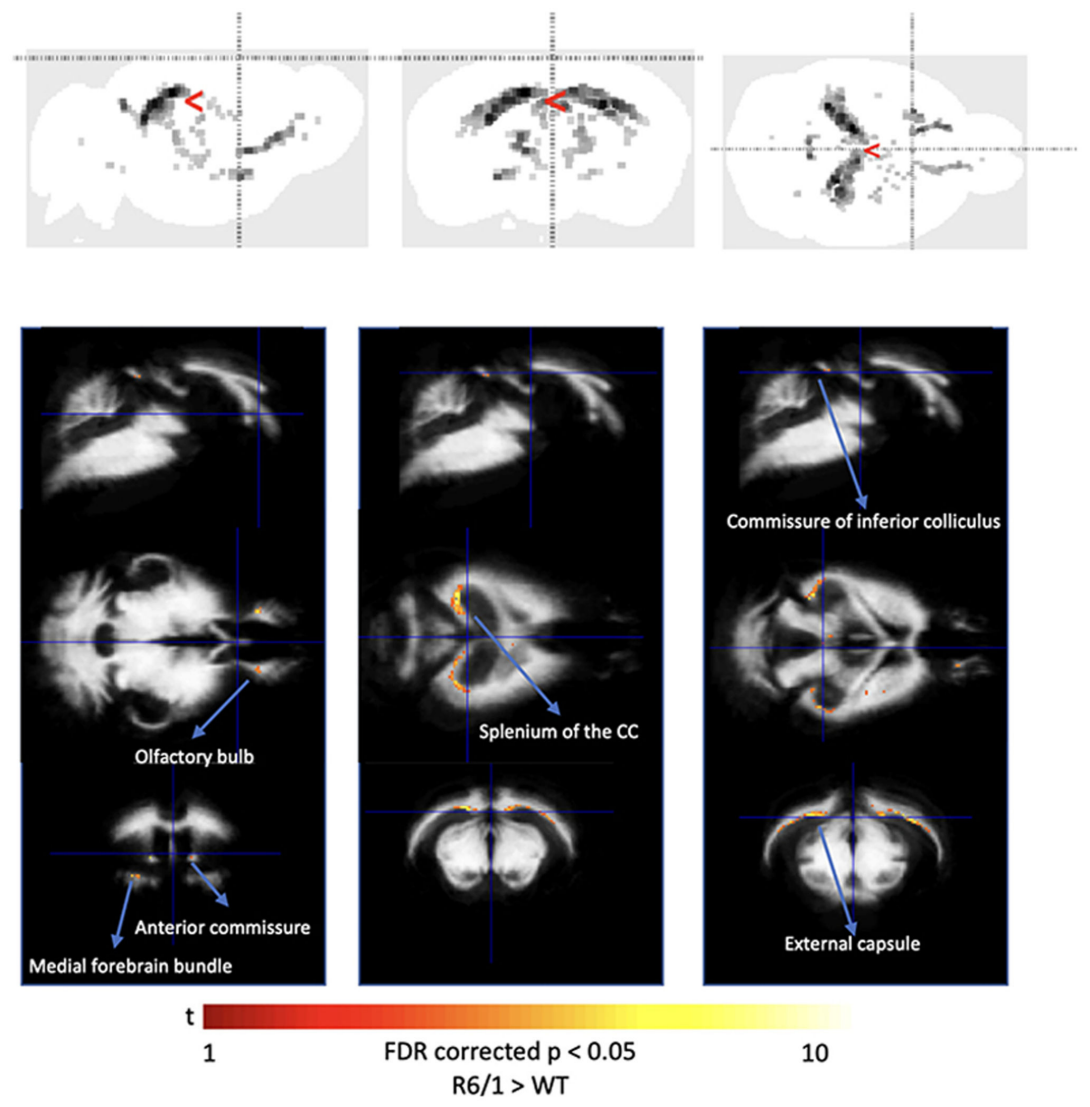
Group-differences in white matter volume. Top: Maximum intensity projections (MIPs). Bottom: Maps of the t-value (whole brain voxel analysis at p < 0.05 False discovery rate-corrected for multiple comparisons) overlayed on template white matter map, demonstrating increased volume in several areas across the brain of R6/1 mice.

Fig. 5 shows regions where white matter volume was significantly higher in R6/1 brains than in WT controls (*p* < 0.05, FDR-corrected). Increased white matter volume was detected in several areas such as the posterior callosum, external capsule and olfactory bulb.

### 3.4 TBSS reveals widespread increases in apparent axon density in R6/1 mice, based on FR, the restricted diffusion signal fraction

**Fig. 6:**
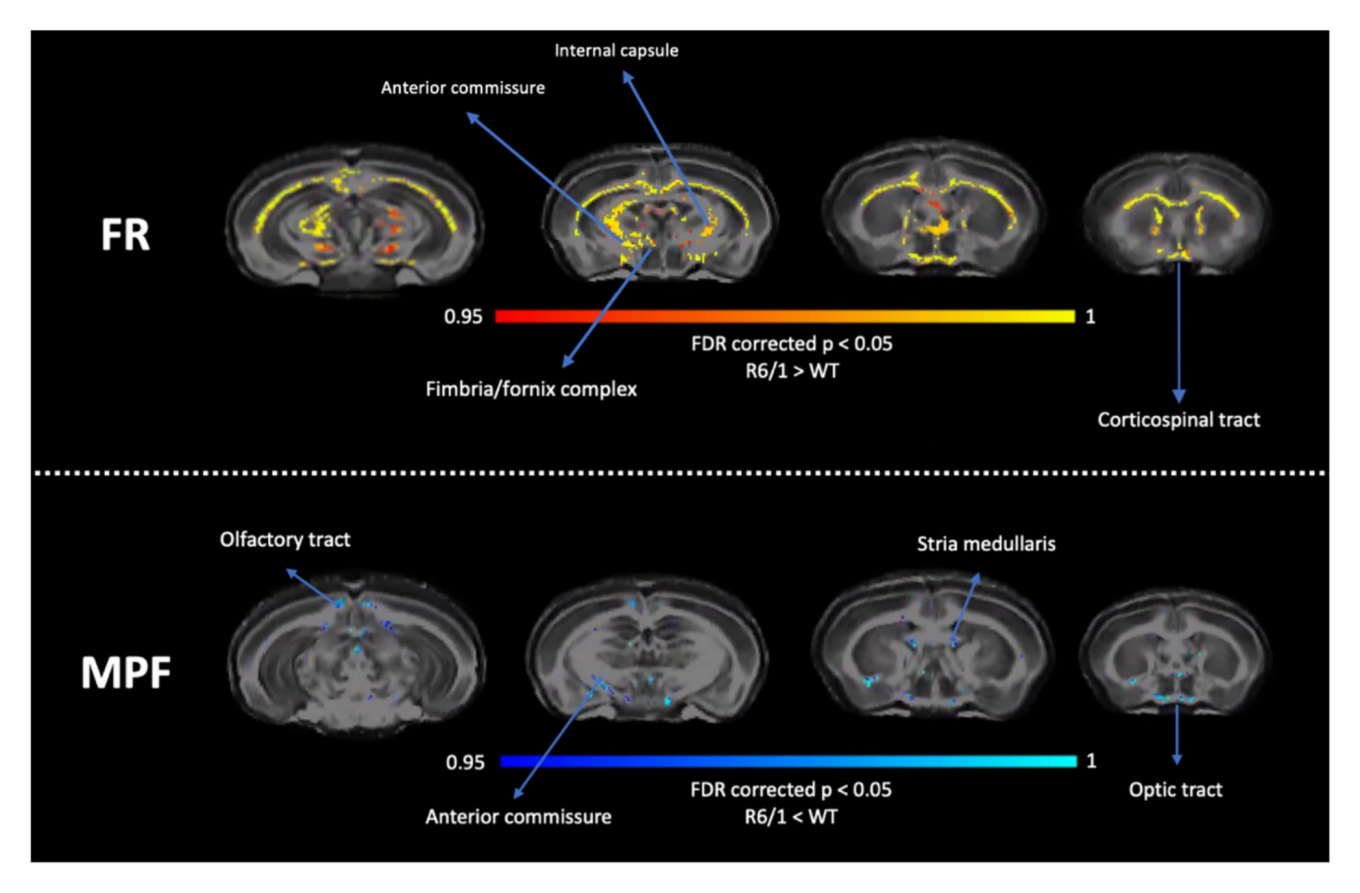
Tract-based spatial statistics (TBSS) analysis of white matter microstructure in R6/1 mice. White matter microstructural alterations were detected across the brain of R6/1 mice, revealing widespread increases in FR and some areas of decreased MPF. Abbreviations: FR: intra-axonal signal fraction. MPF: macromolecular proton fraction. FDR: false discovery rate (Benjamini and Hochberg, 1995).

The TBSS analysis showed widespread increases in FR in the white matter of R6/1 mice (Fig. 6, top) and some decreases in MPF in R6/1 brains (Fig. 6, bottom).

### 3.5 Increased axonal staining and decreased myelin staining in R6/1 mice

**Fig. 7:**
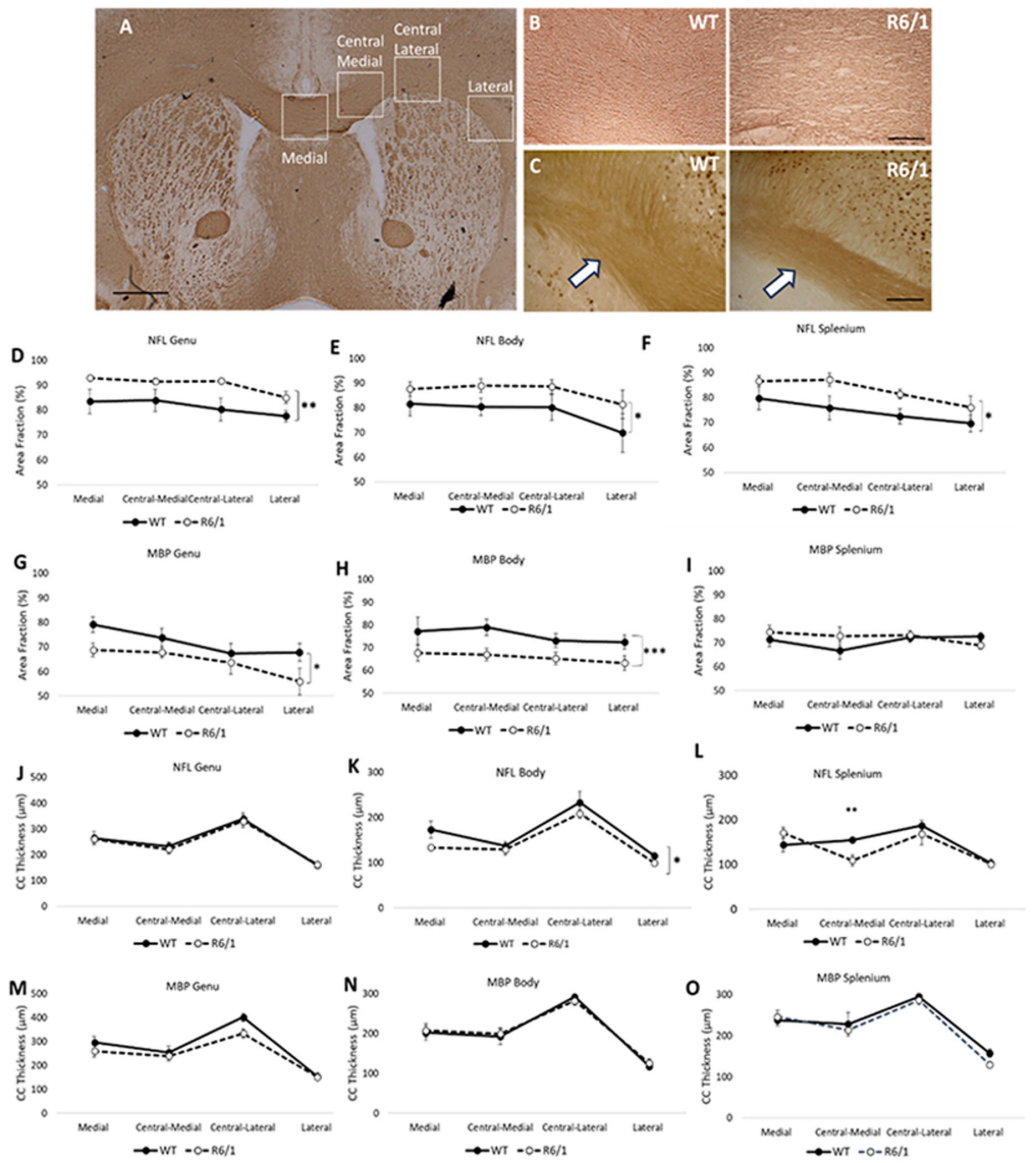
Immunohistochemical analysis of neurofilament light chain (NFL) and myelin basic protein (MBP) in WT (*n* = 9) and R6/1 mice (n = 9). (A) Low magnification (x12.5) MBP immunostaining showing ROIs for analysis along the medial-lateral axis, with one medial ROI and three lateralised ROIs (Central-Medial, Central-Lateral, Lateral). Scale bar = 500 μm. (B) Representative MBP immunostaining at high magnification (x100) in central-lateral region of the corpus callosum body for WT and R6/1 mice. Staining encompasses whole image. Scale bar = 200 μm. (C) Representative NFL immunostaining at high magnification in the central-medial region of the corpus callosum body. White arrows indicate staining in corpus callosum. Scale bar = 200 μm. Analysis of the percentage area stained (area fraction) for the NFL stain within the genu (D), body (E) and splenium (F). Analysis of the percentage area stained (area fraction) for the MBP stain within the genu (G), body (H) and splenium (I). Analysis of the corpus callosum thickness for the NFL stain within the genu (J), body (K) and splenium (L). Analysis of the corpus callosum thickness for the MBP stain within the genu (M), body (N) and splenium (O). Data are presented as mean ± S.E.M. *p ≤ 0.05, **p ≤ 0.01, ***p ≤ 0.001.

Representative images of MBP and NFL immunostaining in wildtype and R6/1 mice (Fig. 7A-C). Across the medial-lateral axis, a higher percentage of corpus callosum area was immunostained for neurofilament light in R6/1 mice, as compared to wild-type mice, in the genu, body and splenium [Fig. 7D-F; Splenium, min. F(1, 16) = 5.00, *p* < 0.05]. Conversely, a lower percentage area was stained for MBP in the R6/1 mice, as compared to wildtype mice, in the genu and body [Fig. 7G-I; Genu, min. (F(1, 18) = 6.03, p < 0.05].

### 3.6 Thinner corpus callosum in R6/1 body based on NFL, but no difference in MBP

For the NFL immunostain, no differences in corpus callosum thickness were observed in the genu [Fig. 7J; F(1,17) = 1.63, n.s.], but the R6/1 mice had thinner corpus callosum measurements in the body across the medial-lateral axis [Fig. 7K; F(1,13) 6.66, *p* < 0.05] and in the central-medial region of the splenium [Fig. 7L; F(1, 16 = 12.36, *p* < 0.01]. No differences in corpus callosum thickness were detected based on the MBP immunostain [Fig. 7M-O].

### 3.7 Corpus callosum consists of thinner axons in R6/1 mice

**Fig. 8:**
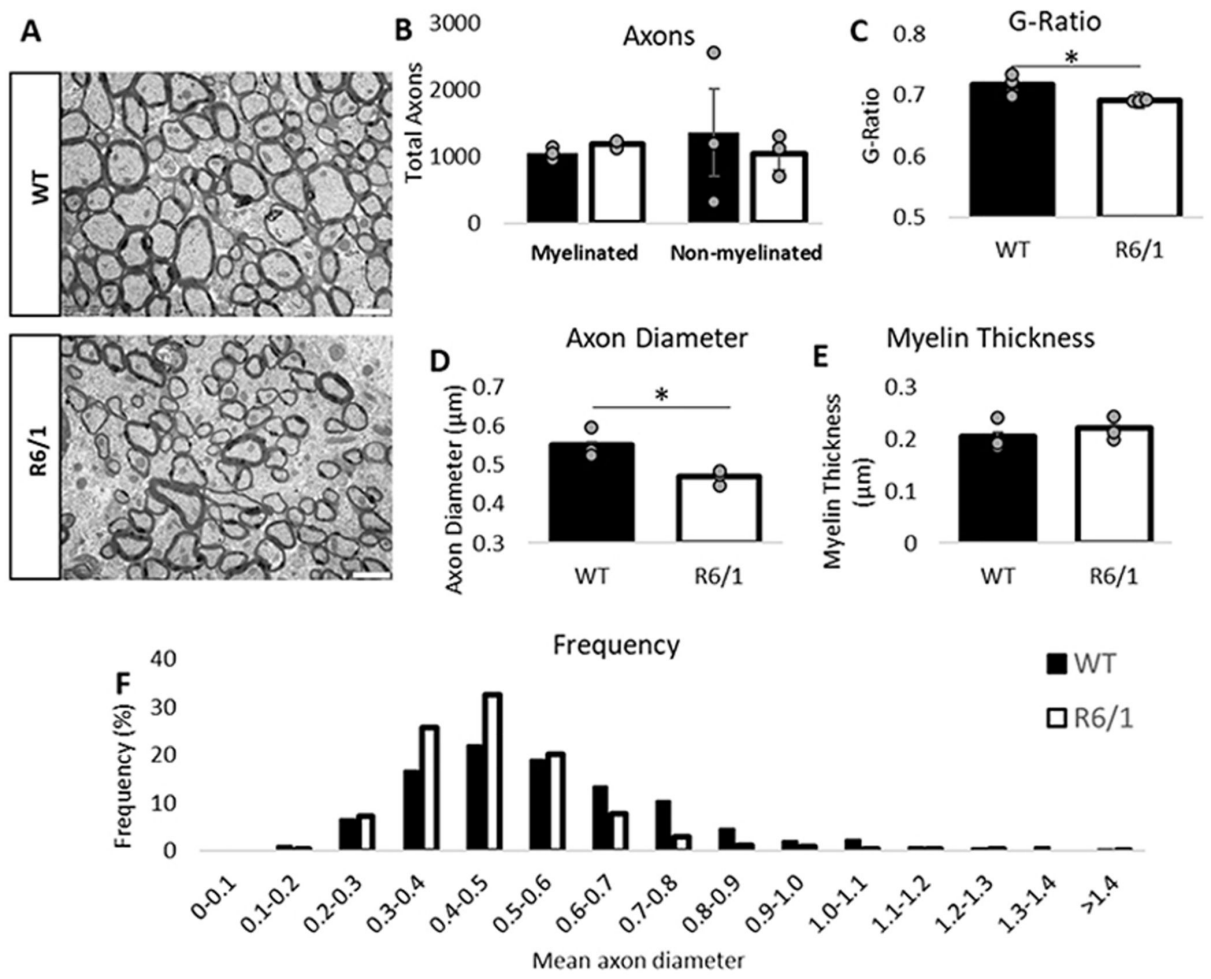
Analysis of axons and myelin via electron microscopy. (A) Representative TEM images from WT (*n* = 3) and R6/1 (n = 3) mice. White scale bars = 1 μm. (B) Total myelinated and non-myelinated axons in wildtype and R6/1 mice. (C) Mean g-ratio measurements for wildtype and R6/1 mice. (D) Mean axon diameter and (E) mean myelin thickness. (F) Percentage frequency of axons measuring 0.0- >1.4 μm in wildtype and R6/1 mice. *N* = 3/group. Data are presented as mean ± S.E.M. *p ≤ 0.05, **p ≤ 0.01, ***p ≤ 0.001.

Representative electron microscopy images from wildtype and R6/1 mice are shown in Fig. 8A. A reduced g-ratio was calculated for R6/1 mice compared to wildtype [Fig. 8C; t(4) = 2.74, *p =* 0.05]. Analysis of the raw data revealed a thinner mean axonal diameter [Fig. 8D; t(4) 3.31, p < 0.05] and this is reflected in the greater frequency of thinner diameter axons in R6/1 mice [Fig. 8F]. No difference in myelin thickness was observed [Fig. 8E; t(4) = −.58, n.s.].

## 4 Discussion

We carried out a high-resolution ex vivo MRI assessment of white matter alterations in the R6/1 mouse model of HD. We assessed 4-month-old mice to represent the early symptomatic stage of the disease (Gatto et al., 2021; Brooks et al., 2012). To explore the cellular causes underlying the imaging phenotype, histological and electron microscopy analyses were performed in a separate cohort of age- and sex-matched mice. Additionally, we investigated the functional correlates of cellular changes by assessing cognitive and motor function in a third age-matched cohort. We report motor and cognitive impairments, as well as novel findings of white matter changes in this model of HD, which may inform future research in the human condition.

### 4.1 Motor and cognitive impairments in R6/1 mice

The motor and cognitive impairments detected in 4-month old R6/1 mice in this study are consistent with previous evidence (Gatto et al., 2021; Brooks et al., 2012). Interestingly, the cognitive deficits in response accuracy in the 5-CSRTT emerged early in training, even when the stimulus duration was long (10s), and the cognitive demands were low. This suggests that rather than an attentional deficit, R6/1 mice manifested a more global visuospatial impairment which disrupts their ability to code their responses in space. Although R6/1 mice were slower to respond, omitted more responses and completed fewer trials, they did not show any impulsivity. Additionally, they showed motor coordination, locomotor activity and dexterity impairments at 4 months old, but muscle strength was not affected. Given this disease-related functional impairment, we used high-resolution ex vivo imaging to explore the neurobiological differences between the genotypes.

### 4.2 White matter microstructural alterations in R6/1 mice

MRI assessment of white matter microstructural changes across the corpus callosum (Jones et al., 2005; Bells et al., 2011) revealed wide-spread increases in FR and decreased MPF in R6/1 mice. Interestingly, TBSS analysis uncovered widespread whole brain increases in FR, together with some regional decreases in MPF, suggesting that such alterations extend beyond the callosum.

It has to be mentioned that the CHARMED model (Assaf and Basser, 2005) utilised to model the diffusion signal in this study represents axons as parallel cylinders and thus cannot recover the effect of axonal orientation dispersion due to bending and fanning of axon bundles widespread throughout the brain. More recent models such as the neurite orientation dispersion and density imaging (NODDI) model (Zhang et al., 2012) extend beyond coherently oriented white matter structures by quantifying the ‘spread’ of neurite orientations within a voxel with the orientation dispersion index (ODI). Notably, a recent ex vivo MRI study by Gatto and colleagues (Gatto et al., 2021) used NODDI to evaluate early microstructural changes in R6/1 mice, and reported increased ODI in the callosal genu at 11 weeks of age. The isotropic volume fraction (IsoVF), representing the signal coming from cerebrospinal fluid and other non-neurite tissue components, was also increased. On the other hand, the intracellular volume fraction (ICVF), representing the fraction of the MRI signal coming from the non-CSF compartment, was lower.

### 4.3 Linking white matter changes to axonal morphology and neurodevelopment

Drawing on both Gatto et al.’s findings (Gatto et al., 2021) and the present results, we suggest that R6/1 mice present early alterations in axonal morphology and structural organization. In line with observations in the R6/2 model (Klapstein et al., 2001), R6/1 mice show a reduction in the overall density of neurites, as reflected by lower ICVF, alongside increased IsoVF and ODI (Gatto et al., 2021). Although total neurite content appears diminished, our observation of elevated FR - mirroring increases previously reported in premanifest patients (Casella et al., 2022) - along with enhanced axonal immunostaining across the callosum, suggests that axonal elements may become relatively more prominent or more tightly packed. One plausible interpretation is that selective loss or retraction of non-axonal neurite compartments (e.g., dendrites) increases the relative contribution and apparent packing density of axons within the remaining tissue volume, thereby giving rise to the higher FR detected in both species.

Notably, we also show that R6/1 mice present axons with disproportionately small diameters, as demonstrated by a thinner mean axonal diameter, and a greater frequency of thinner diameter axons detected with electron microscopy. Although larger-diameter axons typically occupy more space – both because of their calibre and the associated demands of neighbouring glial processes (Barres, 2008; Lamantia and Rakic, 1990; Oberheim et al., 2009) - the present findings indicate that R6/1 mice show a reduction in large-calibre axons rather than an increase in axon density. When fewer thick axons are present, or when axonal morphology is less complex (Gatto et al., 2021; Klapstein et al., 2001), the overall space requirements within the white matter decrease. This altered packing environment can shift the relative contribution of thinner axons without changing the absolute number of axons, leading to an increased proportion of small-calibre fibres. Such shifts in axonal calibre distribution would be expected to elevate FR and contribute to reduced white matter volume (Raven et al., 2023). Overall, this is consistent with our findings of a thinner callosal splenium detected in HD mice with axonal immunostaining, as well as the increased FR detected in the MRI sample.

It is possible that axons in this model develop normally but then degenerate because of the disease process. However, it might also be that R6/1 mice present abnormalities in the postnatal development of axons, and studies evaluating axon microstructure in R6/1 mice earlier in development will be useful in testing this hypothesis. Supporting this possibility, increasing evidence indicates that mHTT disrupts multiple aspects of neurodevelopment well before the onset of overt degeneration. Converging data from rodent models, human stem-cell–derived systems, and imaging studies in mutation carriers – summarised in the review by Humbert and Barnat (Humbert and Barnat, 2022) - show that mHTT affects neuronal differentiation, axonal growth, cytoskeletal organization, synaptic maturation, and early myelination. Such developmental perturbations could plausibly contribute to the thinner axons, reduced morphological complexity, and altered axonal organization observed here. Consistent with a developmental effect of the mutation, typical postnatal maturation is characterised by an increasing proportion of large-calibre axons, whereas thinner fibres predominate earlier in life (Zikopoulos et al., 2018). A disruption of this trajectory by mHTT could therefore shift axonal calibre distributions toward thinner fibres, increasing FR and reducing callosal thickness even before substantial axonal loss occurs.

### 4.4 Myelin alterations and potential implications

In terms of myelin-related alterations in this model, histological analysis revealed that myelin staining was decreased in the genu and body, paralleling MPF decreases observed in the MRI sample. Interestingly, while MBP staining was decreased, electron microscopy did not detect significant alterations in the thickness of myelin sheaths in R6/1 mice.

Electron microscopy primarily focuses on the physical structure of myelin, such as the compactness of myelin layers, while MBP staining specifically targets the presence of MBP, which is a major component of myelin. This in turn suggests that, at least early in disease progression, R6/1 present a reduction in the expression or content of myelin-associated proteins, without significant alterations in the structure of myelin sheaths themselves. Such reductions might in turn be due to changes in gene expression, protein synthesis, or protein degradation.

### 4.5 Inflammation as a potential driver of white matter changes

Importantly, we detected increased white matter volume in R6/1 mice with VBM. Interestingly, such an increase also concerned the splenium of the callosum, where NFL staining detected reduced thickness. Although these findings might seem counterintuitive, it is important to stress that VBM and NFL staining have different spatial resolutions and sensitivities. Specifically, while NFL staining provides fine-grained information about specific axonal changes, VBM captures broader structural changes that include factors other than axons. Therefore, an increase in volume as detected by VBM could be due to changes in the size or quantity of brain tissue components other than axons, such as glial cells, blood vessels, or inflammation-related oedema and swelling.

Neuroinflammation is one of the central mechanisms involved in HD-related pathology, and reactive inflammatory processes are one of the most studied at the early stages of HD (Politis et al., 2015). Consistent with this, Gatto et al. (Gatto et al., 2021) demonstrated the presence of neuroinflammatory processes already at the early stages of disease in R6/1 mice. Therefore, swelling of glial cells may have biased brain atrophy measurements (Andravizou et al., 2019) and may underlie the volume increases observed in this study. Inflammation-related swelling of glia may also explain the widespread increases FR observed in R6/1 mice, by restricting diffusion in the extra-axonal compartment (Arfanakis et al., 2002; Stidworthy et al., 2003).

Although we did not initially explore neuroinflammatory changes in the brains of these mice, these findings prompted an analysis of astrocytes (GFAP) and microglia (Iba1) in the splenium, using the remaining immunohistochemical tissue (WT: *n =* 5; R6/1: n = 5). This revealed a trend toward increased microglial expression in the splenium (Supplementary Fig. 2) in R6/1 mice, which is consistent with the literature. Given these indications of early inflammatory involvement, it is informative to consider how inflammation-related processes may influence diffusion MRI metrics in this model.

The diffusion findings from Gatto et al. (Gatto et al., 2021) provide important context for interpreting these results. While they reported a modest reduction in ICVF at 11 weeks, the more prominent increase in IsoVF was attributed to inflammation-related elevations in interstitial free water. This distinction is important in the context of our work because the CHARMED model used here does not include an explicit free-water compartment. As a result, any signal contributions arising from inflammatory processes - such as increased cellularity from activated microglia or astrocytes, or restricted interstitial water - are incorporated into FR rather than separated out. Consequently, FR in our dataset reflects both neurite-specific features and inflammation-associated sources of restricted diffusion. Depending on the balance between neurite loss, axonal calibre changes, and inflammatory responses, FR may therefore increase, decrease, or remain stable. Differences in disease stage between studies (11 weeks in Gatto et al. vs. 16 weeks in the present work) may also contribute to the divergence observed between fICVF and FR.

### 4.6 Translational implications and biomarker potential

To summarise, we report differences in white matter microstructure in the R6/1 mouse model of HD. By combining insight from MRI, histology, and electron microscopy, as well as evidence from previous studies, we suggest that such alterations are likely driven by axonal changes. Specifically, we show that R6/1 mice present disruptions in axonal morphology and organization. Furthermore, we show that, at least early in disease progression, R6/1 present a reduction in the expression or content of myelin-associated proteins, without significant alterations in the structure of myelin sheaths themselves. Finally, our findings indicate that neuroinflammation-driven glial and axonal swelling might also affect this mouse model early in disease progression.

These results extend previous findings on animal models and HD patients, all of which demonstrate alterations in white matter micro-structure as a significant feature of HD (Casella et al., 2021; Casella et al., 2022; Jin et al., 2015; Meng et al., 2017; Tabrizi et al., 2011; Rosas et al., 2010; Bourbon-Teles et al., 2019; Di Paola et al., 2012; Crawford et al., 2013; Johnson et al., 2021; Zhang et al., 2018). Interestingly, recent diffusion-MRI work in the heterozygous zQ175DN knock-in model (Vidas-Guscic et al., 2024) has demonstrated early reductions in fibre density and cross-section before overt neurodegeneration, occurring in the absence of major myelin loss. These findings strongly parallel the early microstructural alterations we observed in R6/1 mice - namely, increased FR, reduced MBP content, and axonal thinning on EM - suggesting that disruptions to axonal morphology and organization represent an early and conserved feature across distinct HD mouse models. Importantly, the convergence between knock-in and fragment-transgenic models reinforces the interpretation that FR is sensitive to axonal structural alterations rather than demyelination, and supports its promise as a translational biomarker, consistent with our prior demonstration of increased FR in premanifest HD patients (Casella et al., 2022).

Biomarkers constitute an important tool of translational science in medicine, thus the development of biomarkers that are useful and accessible in both animal models and humans affords improved translation of animal findings into humans and, likewise, the back-translation of imaging methods from humans to animal models (Wendler and Wehling, 2010). Therefore, while inherent differences between species remain, the present findings may represent an important preliminary step in the establishment of FR as a novel imaging biomarker of white matter pathology in HD.

### 4.7 Methodological considerations

We worked with female WT and R6/1 mice throughout this study, for pragmatic reasons related to available resources and to limit any variability potentially introduced by mixing male and female mice without increasing the ‘n’/power. Ideally, we would have worked with box sexes, to ensure that the effects we report are relevant to both males and females. There are limited data suggesting sex differences in weight and social behaviours in R6/1 mice (Harrison et al., 2013; Pietropaolo et al., 2011; Brooks et al., 2012), with most studies not testing or not reporting sex differences, which is an inherent limitation.

The MRI data were obtained ex vivo from fixed tissue. As several factors, such as tissue fixation, sample temperature, and the acquisition scheme all have an effect on MRI measures, considerable care should be taken when extrapolating the present ex vivo findings to in vivo values (Antonsen et al., 2013). However, the observed group differences should be valid, since any bias in terms of methodology should have the same effect on the two groups compared. Furthermore, in the present study, several steps were taken with regards to the perfusion and tissue preparation protocols to obtain optimal tissue quality. Such procedures were based on previous literature (Cahill et al., 2012), and consisted of the utilisation of liquid, rather than powder, perfusates, as well as delivery of perfusate at a low flow rate, in order to avoid blockage of the capillary beds and vessels’ rupture. Additionally, the storage of tissue in a sodium azide solution enabled improved tissue conservation. Another important thing to consider is that this study assessed distinct cohorts of mice. Therefore, the nature of the study design limits our ability to establish causal relationships between variables.

Overall, our study provides an important stepping stone for future research assessing axonal alterations as a pathophysiological feature of HD. These findings are in line with broader evidence from other rodent HD models. A recent review has synthesized MRI findings across a range of preclinical models, highlighting that microstructural alterations in white matter are a common feature, though their onset and severity may vary depending on genetic background and disease stage (Zhang et al., 2012). Our data contribute to this understanding by demonstrating, in R6/1 mice, a reduction in the expression or content of myelin-associated proteins without significant alterations in the structure of myelin sheaths. Crucially, our findings highlight the potential of FR, an in vivo estimate of axon density, as a novel MRI biomarker of HD-associated changes in white matter microstructure.

## Supplementary Material

Supplementary data to this article can be found online at https://doi.org/10.1016/j.nbd.2026.107318.

Supplementary Data

## Figures and Tables

**Table 1:** Summary of assessments carried out in this study and sample description.

Sample description	Assessment
7 WT and 8 R6/1 female mice tested at 4 months of age	Ex vivo MRI: ◼ Tractometry of the corpus callosum (FA, AD, RD, FR and MPF) ◼ TBSS (FA, AD, RD, FR and MPF) ◼ VBM (anatomical data)
6 WT and 5 R6/1 female mice tested from 1 to 4 months of age	Assessment of cognitive function: ◼ 5-CSRTT Assessment of motor function: ◼ Fixed rotarod ◼ Accelerated rotarod ◼ Vertical pole ◼ Locomotor activity ◼ Grip strength
9 WT and 9 R6/1 female mice tested at 4 months of age	Light microscopy: ◼ NFL and MBP corpus callosum thickness and area fraction
3 WT and 3 R6/1 female mice tested at 4 months of age	Electron microscopy: ◼ Axon diameter of myelinated axons ◼ G-ratio of myelinated axons

**Table 2:** Scan parameters. All sequences were acquired at 9.4 Tesla. For each of the sequences, the main acquisition parameters are provided. MT: magnetization transfer; qMT: quantitative magnetization transfer. FoV: field of view; TE: echo time; TR: repetition time.

MRI acquisition parameters description	
Anatomical scan	
Pulse sequence	FLASH (3D)
Matrix size	192 × 192 × 72
FoV	19.2 × 19.2 × 18
TE, TR (ms)	4, 30
Flip angle (°)	36
Acquisition duration	20 min 44 s
DTI/CHARMED	
Pulse sequence	RARE
Matrix size	192 × 192
FoV	19.2 × 19.2
b-values (s/mm^2^) – gradient directions	0, 1200–50, 2400–50, 4000–50
δ/α (ms)	6.8/16.3
Slice number/thickness (mm)	20/0.4
TE, TR (ms)	31, 3000
Flip angle (°)	90
Partial Fourier	1
acceleration factor	
Acquisition duration	12 h 14 min
T_1_ map	
Pulse sequence	FLASH (3D)
Matrix size	192 × 192 × 72
Fov	19.2 × 19.2 × 18
TE, TR (ms)	4, 30
Flip angles (°)	13, 17, 24, 36, 48
Acquisition duration	1 h 45 min
MT/ qMT	
Pulse sequence	FLASH 3D
Matrix size	192 × 192 × 72
FoV	19.2 × 19.2 × 18
TE, TR (ms)	5, 47
Off-resonance pulses (Hz/°)	0/350, 1000/350, 1500/350, 3000/350, 6000/350, 12000/350, 24000/350, 1000/950, 1500/950, 3000/950, 6000/950, 12000/950, 24000/950
Flip angles (°)	5
Acquisition duration	9 h 50 min

## Data Availability

The data analysed during the current study and the respective analysis scripts are available from the corresponding author on reasonable request.
