# Supplementary material for: Differences in white matter detected by ex vivo 9.4 T MRI are associated with axonal changes in the R6/1 model of Huntington’s disease
Source: Neurobiol Dis. Author manuscript; Available in PMC 2026 Jun 13. (PMC7619152; doi:10.1016/j.nbd.2026.107318)
Supplement: Supplementary Data [file EMS214117-supplement-Supplementary_Data_.docx]

**Supplementary Information**

***Supplementary Figure 1. Quality control of imaging data.*** *Representative MRI images showing examples of excluded (top row) and included (bottom row) datasets based on quality control criteria. The excluded datasets exhibited perfusion-related artifacts, including air bubbles (indicated by red arrows), which caused localized signal voids and distortions in the imaging data. In contrast, the included datasets maintained clear structural boundaries and uniform signal intensity, ensuring reliable measurements for further analysis.*

**
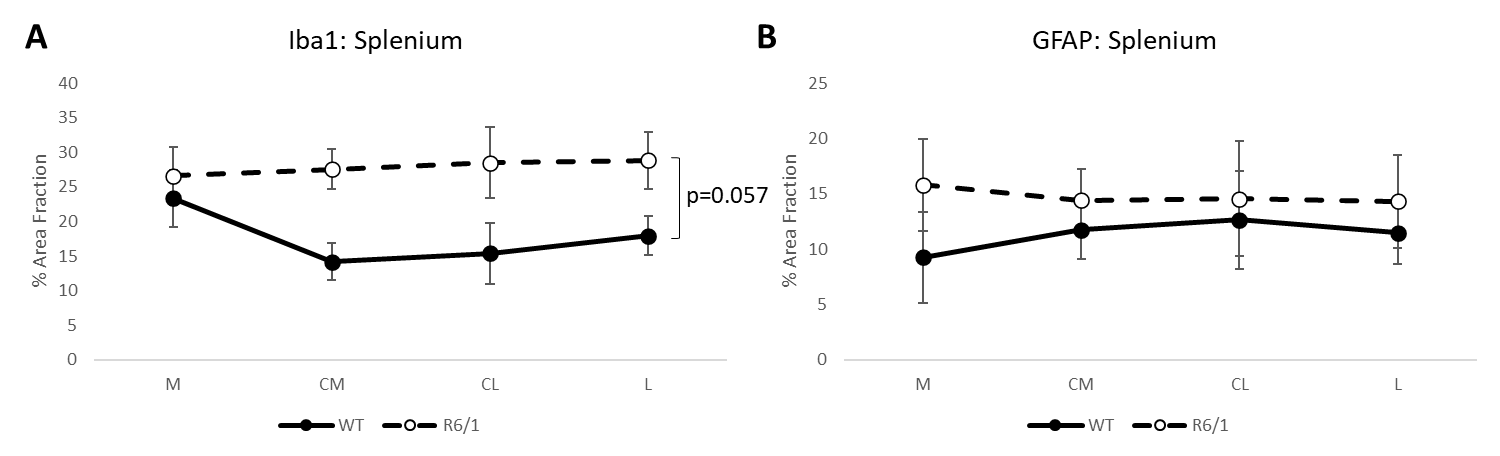
**

***Supplementary Figure 2:*** *Immunohistochemical staining for microglia (Iba1) and astrocytes (GFAP) in WT (n=5) and R6/1 (n=5) splenium. (A) There was a trend for more microglia in the R/1 brain [Genotype: F_1,8_=4.95, p=0.057]. There were no interactions between Genotype and Region [F_3,24_=1.78, p=n.s.], nor an effect of Region [F_3,24_=1.02, p=n.s.]. (B) No differences in astrocytes were evident [Genotype: F_1,8_=2.20, p=0.057; Genotype*Region [F_3,24_=2.13, p=n.s.], Region [F_3,24_=0.40, p=n.s.]. Errors bars: ±S.E.M.*
